# Inverse Association between Obesity Predisposing *FTO* Genotype and Completed Suicide

**DOI:** 10.1371/journal.pone.0108900

**Published:** 2014-09-29

**Authors:** Izabela Chojnicka, Sylwia Fudalej, Anna Walczak, Krystyna Wasilewska, Marcin Fudalej, Piotr Stawiński, Katarzyna Strawa, Aleksandra Pawlak, Marcin Wojnar, Paweł Krajewski, Rafał Płoski

**Affiliations:** 1 Department of Medical Genetics, Medical University of Warsaw, Warsaw, Poland; 2 Department of Rehabilitation Psychology, University of Warsaw, Warsaw, Poland; 3 Department of Psychiatry, Medical University of Warsaw, Warsaw, Poland; 4 Department of Forensic Medicine, Medical University of Warsaw, Warsaw, Poland; 5 Department of Psychiatry, University of Michigan, Ann Arbor, Michigan, United States of America; University of Vienna, Austria

## Abstract

The A allele of rs9939609 in the *FTO* gene predisposes to increased body mass index (BMI) and obesity. Recently we showed an inverse association between the obesity related A allele of rs9939609 and alcohol dependence which was replicated by others. Since this finding raises a possibility that *FTO* may be associated with other psychiatric phenotypes, we aimed to examine association of rs9939609 with completed suicide. We genotyped rs9939609 in 912 suicide victims and 733 controls using TaqMan approach. We observed an inverse association between suicide and the rs9939609 A allele (*OR* = 0.80, *P* = 0.002, *P_cor_* = 0.006) with genotype distribution suggesting a co-dominant effect. Given the link between alcoholism and suicide under influence of alcohol reported in Polish population, confounding by alcohol addiction was unlikely due to apparently similar effect size among cases who were under influence of ethanol at the time of death (*OR* = 0.76, *P* = 0.003, *N* = 361) and those who were not (*OR* = 0.80, *P* = 0.007, *N* = 469). The search for genotype-phenotype correlations did not show significant results. In conclusion, our study proves that there is an inverse association between rs9939609 polymorphism in *FTO* gene and completed suicide which is independent from association between *FTO* and alcohol addiction.

## Introduction

The *FTO* gene and particularly the A allele of rs9939609 polymorphism is known for association with body mass index (BMI), obesity risk, and type 2 diabetes [1], which has been confirmed in a Polish population [2] as well as in a recent multiethnic study including over 170 000 individuals [3].

The exact function of *FTO* gene, mainly expressed in several brain regions [4] including pineal, prefrontal cortex, hypothalamus and amygdala (http://biogps.org/), is not known. *FTO* has been suggested to play a role in the control of food intake and food choice predisposing to a hyperphagic phenotype or a preference for energy-dense foods [5]–[7] possibly due to modulation of the CREB signaling pathway [8].

There is evidence that *FTO* variants are associated not only with BMI, but with psychological/psychiatric phenotypes as well. The effect of *FTO* on BMI has been shown to be influenced by history of depression [9] or the antipsychotic treatment [10]. Moreover, *FTO* polymorphisms were associated with depression/anxiety [11] and psychological distress [12]. On the other hand, a recent study has shown that *FTO* rs9939609 A variant, which predisposes to obesity, has a protective effect on depression [13]. Yet, the study in a South Australian population found no differences between values of BMI in completed suicide victims and controls who died in other way [14]. Our previous study showed a link between the *FTO* gene and alcohol drinking habits among general population as well as an inverse association of the obesity associated variant with alcohol dependence [2]. Recently, this protective effect of *FTO* variants on alcoholism has been confirmed by independent studies in other populations [15], [16].

It is well known that the most prevalent risk factors for committing suicide are mental and psychiatric disorders [17]. Given the reported links between *FTO* polymorphism and psychiatric phenotypes we speculated that *FTO* variant rs9939609 could also be associated with suicide. The aim of the study was to verify this hypothesis.

## Materials and Methods

### Subjects

Suicide victims were consecutive cases from Warsaw metropolitan area autopsied in the Department of Forensic Medicine at the Medical University of Warsaw, Poland. The assignment of the cause of death to an act of suicide was based on the results of the autopsy and the circumstances of death. The autopsy was undertaken to determine the cause of death, including the characteristic features of a particular type of suicide (e.g. in case of hanging the typical ligature marks on the neck, their intravitality, evidence of pressure on the neck, absence of potential signs of struggle and the toxicological analysis of the biological material). The final decision about the classification of a particular case as suicide was made by the prosecutor on the basis of autopsy results, evidence collected at the place where the body was found and the testimony of witnesses.

We collected the information about clinical variables, such as age, gender, suicide method and blood ethanol concentration from the post-mortem medical and forensic examination protocols. According to legal regulations in Poland blood ethanol concentration (BAC)>0.2 mg/dl was considered as indicative of being under influence of alcohol.

The samples (*N* = 942) were collected between 2005 and 2013. Characteristics of suicide victims is shown in Table 1. The control group (*N* = 747) came from a previously described group of adult subjects representative of Central Poland population [2]. The study was approved by the Bioethical Committee of the Medical University of Warsaw and all control subjects gave written consent for the anonymous use of their DNA for research. All the data used in the study was anonymized. All cases and controls were Polish Caucasians.

**Table 1:** Characteristics of suicide subjects.

Variable		Number of cases (%)
**Gender**	Males	739 (83.31)
(data available for 887 cases)	Females	148 (16.69)
**Age** (data available for 714 cases)	mean = 43.18, *SD* = 16.69	714
**Blood ethanol concentration** (data available for 850 cases)	Under influence of alcohol (ethanol concentration>0.2 mg/dl), mean = 1.79, *SD* = 1.05	365 (42.94)
	Without influence of alcohol (ethanol concentration ≤0.2 mg/dl)	485 (57.06)
**Method of committing suicide** (data available for 854 cases)	Hanging	664 (77.75)
	Jumping from a high place	97 (11.36)
	Self-harm by sharp object	22 (2.58)
	Shot with a firearm	24 (2.81)
	Jumping or lying before moving object	14 (1.64)
	Toxic effect from ingested substance	17 (1.99)
	Other	16 (1.87)

### Genotyping

Genomic DNA from cases and from controls was isolated from EDTA blood samples using standard salting out procedure [18].

Genotyping of the *FTO* variant rs9939609 was performed by Real-time TaqMan Allelic Discrimination Assay using pre-designed primers obtained from Applied Biosystems (Pre-designed TaqMan SNP Genotyping Assays, 7500 Real time PCR System, Applied Biosystems, Assay ID C__30090620_10) on ABI PRISM 9700 platform (Applied Biosystems).

We genotyped 874 completed suicide subjects (+68 undetermined samples, 93% call rate). As the call rate was low, we purified 43 undetermined samples containing enough DNA using Agencourt AMPure XP (Beckman Coulter) which allowed to successfully genotype additional 38 samples bringing the total number of samples successfully genotyped to 912 (call rate 97%). Among controls 733 subjects were successfully typed (14 undetermined genotypes, 98% call rate).

### Statistical analysis

Distribution of rs9939609 alleles and genotypes among cases and controls was compared using a chi square test using Statistica software version 10.0 (StatSoft, Inc.Tulsa). Genotype analyses were performed assuming dominant, co-dominant or recessive models using Web-Assotest program (http://www.ekstroem.com/assotest/assotest.html), where co-dominant model, also known as additive or multiplicative penetrance model, describes the situation in which each copy of the risk allele increases the risk linearly. The most likely model of inheritance was determined by *P* value for model fit (*P_fit._*), which allows to estimate whether given model is consistent with the distribution of genotypes (*P*_fit_<0.05 indicates that given model should be rejected). *P_fit_* was calculated by a Chi square test. The strength of associations was assessed by *Odds Ratio* (*OR*). Search for genotype-phenotype correlation among suicides was performed using chi square test, linear regression or Student's t test implanted in Statistica software version 10.0 (StatSoft, Inc.Tulsa, OK).

Our study had the power of 0.8 to detect at alpha = 0.05 an effect associated with allelic *OR* = 0.8.

Due to multiple testing, we used Bonferroni correction. In the analysis of the model of inheritance, three models were analyzed, thus the correction factor was three. Genotype-phenotype correlations were tested only for one, most probable model, therefore, the *P*-values were corrected for the number (N = 3) of analyzed phenotypes, i.e. sex, age and blood alcohol concentration. The Bonferroni corrected *P*-values are indicated as *P_cor_*.

## Results

### The A allele of rs9939609 is inversely associated with completed suicide

The distribution of genotypes was in Hardy-Weinberg Equilibrium (HWE) among cases (*P* = 0.617) and controls (*P* = 0.326). We observed lower frequency of the rs9939609 A allele among cases (42%) vs. controls (47%, *OR* = 0.80, *P* = 0.002, Table 2). The genotype distribution indicated that both the co-dominant and recessive models were the plausible models of inheritance, although the former had the best fit (co-dominant: *OR* = 0.81, *P* = 0.002, *P_cor_* = 0.006, *P_fit_* = 0.70; recessive: *OR* = 0.72, *P* = 0.008, *P_cor_* = 0.024, *P_fit_* = 0.117).

**Table 2:** Distribution of genotypes and analysis of the association between rs9939609 and suicide.

	rs9939609 genotypes				Model		
				Allelic comparison*	Recessive*	Co–dominant*	Dominant*
				OR (CI), *P*	OR (CI), *P* [*P_cor_*], *P_fit,_*	OR (CI), *P* [*P_cor_*], *P_fit_*	OR (CI), *P* [*P_cor_*], *P_fit_*
	TT (%)	AT (%)	AA (%)		(AA vs. TT/AT)	(AA vs. AT vs. TT)	(AT/AA vs. TT)
Suicide victims (*N* = 912)	314 (34.43)	436 (47.81)	162 (17.76)	0.80 (0.70; 0.92), **0.002**	0.72 (0.57; 0.92), **0.008 [0.024]**, 0.117	0.81 (0.70; 0.93), **0.002 [0.006]**, 0.700	0.77 (0.63; 0.96), **0.017** [0.051], 0.051
Suicide victims with blood ethanol concentration>0.2 mg/dl (*N* = 361)	133 (36.84)	164 (45.43)	64 (17.73)	0.76 (0.64; 0.91), **0.003**	**–**	0.77 (0.65; 0.92), **0.004 [0.012]**, 0.732	**–**
Suicide victims with blood ethanol concentration ≤0.2 mg/dl (*N* = 469)	163 (34.75)	223 (47.55)	83 (17.70)	0.80 (0.67; 0.94), **0.007**	**–**	0.80 (0.68; 0.94), **0.008 [0.024]**, 0.800	**–**
Controls (*N* = 733)	212 (28.92)	352 (48.02)	169 (23.06)				

*P* values <0.05 are **boldfaced**; *P_cor_* – *P* values corrected for multiple testing with Bonferroni method;

* comparison with controls.

### The association of the rs9939609 A allele with completed suicide vs. association with alcohol addiction

Since in Polish population there is a strong association between alcoholism and suicide committed under influence of alcohol [19] we compared distribution of genotypes between cases with blood alcohol concentration (BAC) over 0.2 mg/dl and cases with BAC equal to or lower than 0.2 mg/dl. We found no significant differences in these comparisons (*P* = 0.99).

Furthermore, when suicides with BAC< = 0.2 mg/dl were compared to controls under co-dominant model, a decreased prevalence of the rs9939609 A allele was found (*OR* = 0.80, *P* = 0.008, *P_cor_* = 0.024, Table 2).

### Exploratory search for rs9939609 genotype-phenotype correlations

We found no differences in rs9939609 allele distribution between male and female suicide cases (*P* = 0.284) as well as no association between rs9939609 genotype and age (*R^2^* = 0.0023, *P* = 0.203).

## Discussion

The major novel finding in our study is the inverse association between the obesity associated A allele of rs9939609 in the *FTO* gene and completed suicide. No confounding effect of alcohol addiction in our study is supported by the lack of difference in *FTO* allele/genotype distribution between cases who were under influence of ethanol at the time of death and those who were not. If our results were secondary to the described link between *FTO* variants and alcoholism such an association could be expected as we have previously shown that in Polish population alcoholism and suicide under influence of alcohol frequently co-occur [19]. Furthermore, BAC did not correlate with *FTO* genotype and the association with rs9939609 was found both among those with increased BAC and those with BAC<0.2 per mille.

Whereas the association between suicide and *FTO* has not been studied before, two recent studies searched for correlation between *FTO* genotype and psychological traits. In the Copenhagen General Population Study of 53,221 adults the obesity predisposing *FTO* allele was negatively associated with psychological distress [12]. These results are directly consistent with our findings that the obesity related *FTO* rs9939609 allele/genotype is less prevalent among suicides than in general population.

Conversely, the other study described a positive association between the obesity related *FTO* variant and depression/anxiety which was present among 2,981 men but not 1,164 women from population of The Whitehall II Study [11]. The reasons for these discrepant results are not clear [20] and may indicate population specific differences. It should be noted that Kiwamaki et al. failed to observe the well documented association between *FTO* and BMI in females suggesting that the studied population might have been biased in some way [11].

The A allele of rs9939609 in the *FTO* (and/or other tightly linked variants) are strongly associated with BMI and obesity, also in the Polish population [2]. Interestingly, the association between BMI and psychological/psychiatric traits is a subject of controversy with the two opposing views formulated as the “jolly-fat” and “fat-sad” hypotheses. The “jolly-fat” hypothesis is based on reports of reduced risk of psychological distress and mental disorders, particularly psychoses, mood, and anxiety disorders, among obese persons [12], [21]–[23]. The “fat-sad” theory posits the opposite and is supported by a recent meta-analysis [24]. As we did not assess BMI in our subjects, it is not clear, whether the observed association of *FTO* rs9939609 with completed suicide is independent of BMI or not. We favor the hypothesis that *FTO* acts on mood and suicide in other ways than through weight gain which is regarded as modest (2–3 kg). Such an indirect effect would be consistent with our previous observations that the link between rs9939609 and alcohol dependence was independent of BMI [2].

In the last three years 4 genome-wide association studies (GWAS) have been published on attempted or completed suicide, suicidal ideation, thoughts or behavior [25]–[28] and none of these reported any *FTO* polymorphism as having a significant association with suicidility. Nevertheless, it does not mean that rs9939609 has no effect whatsoever, as GWAS typically have low power to detect the association on a single variant level.

*FTO* is a member of family of m^6^A RNA demethylases and recently methylation of the N6 position of adenosine (m^6^A) in RNA has been shown to be markedly increased throughout brain development [29]. Whereas direct effect of rs9939609 on *FTO* function is possible, recently it has been suggested that at least for obesity the associated *FTO* region exerts its effect modulating expression of another relatively distant gene - *IRX3* which encodes a transcription factor highly expressed in brain [30]. To investigate this, the authors examined *IRX3* expression in 153 human brain samples. Obesity-linked SNPs were associated with *IRX3* expression in these samples, but not with expression of *FTO*, directly linking these variants to *IRX3* regulation. However, as Cedernaes and Benedict conclude in their commentary [31], further studies are needed to determine whether the associations reported so far are caused by a direct link with *IRX3* or *FTO* or both. Regardless of the precise mechanism and provided our results are replicated in other populations, our data indicate that the variation in the rs9939609 region has important effects on psychiatric phenotypes which extend beyond its influence on body weight.
